# Supplementary material for: Low self-esteem in adolescents with sickle cell disease: Determinants and coping mechanisms; A mixed methods study
Source: PLoS One. 2026 Mar 12;21(3):e0344864. doi: 10.1371/journal.pone.0344864 (PMC12981461; doi:10.1371/journal.pone.0344864)
Supplement: Attachment — Submitted filename: Rebuttal_letter.docx [file pone.0344864.s002.docx]

**Makerere university**

**Department of pediatrics and child health**

**8^th^ December 2025**

**Academic Editor**

**PLOS one**

**RE: Rebuttal letter for manuscript**

| **S/No** | **Comment** | **Response** |
| --- | --- | --- |
|  | **Academic editor** | |
|  | 1) Title, authors, affiliations, and front matter  • Title (remove place of study): The checklist asks to delete the place of the study from the title. Recommend:  o Proposed title: “Low Self-Esteem in Adolescents with Sickle Cell Disease: Determinants and Coping Mechanisms — A Mixed-Methods Study.”  • Affiliation formatting & consistency: Use only “Department, University, City” (capitalize proper nouns; singular/plural consistently). E.g., “Department of Pediatrics and Child Health, Makerere University College of Health Sciences, Kampala, Uganda.” Ensure the two affiliations follow the same pattern. Fix capitalization (“Makerere University,” not “Makerere university”).  • Name inconsistency: “Grace Ndeezi” vs “Grace Ndezi” (one ‘e’ missing in the author line). Standardize to the correct spelling throughout.  • Corresponding author details: Email and phone are present; keep only in the author block (do not repeat elsewhere).  • Numbering of sections: Use journal-consistent numbering (e.g., 1. Introduction; 2. Materials and Methods; 3. Results; 4. Discussion; 4.1 Limitations; 5. Conclusion).  2) Abstract  • Structure: Generally appropriate (Background, Methods, Results, Conclusion).  • Sample size placement: Good—sample size is in Methods (n=356).  • Effect directions & precision: Clarify directionality. As coded in the model, “No” school absence and “No” physical limitation are protective (PRs < 1), while limited social engagement increases prevalence (PR > 1). Rewrite to avoid ambiguity like “included school absenteeism” without direction. Suggest:  o “Lower prevalence of low self-esteem was observed among adolescents not frequently absent from school (aPR = 0.94, 95% CI 0.90–0.99) and those without limitations to physical activity (aPR = 0.94, 95% CI 0.90–0.98), while limited social engagement was associated with higher prevalence (aPR = 1.16, 95% CI 1.03–1.31).”  • Statistics in abstract: Keep measures (PRs, CIs, p-values) but don’t mention software names here (that belongs in Methods).  3) Introduction  • Currency & concision: Reasonable framing; tighten historical/global figures to 1–2 citations and remove duplication.  • Terminology consistency: Use THRIVE (all caps) consistently (appears as “Thrive/THRIVE” in different places).  • Aim statement: Place the aim at the end of the Introduction as a final sentence, not as a separate paragraph.  4) Materials and Methods  • Design & setting: Clear mixed-methods, sequential explanatory; dates and clinic context included. Good.  • Sample size inconsistency: Text states “Three hundred and fifty-seven (356)”—fix to 356 everywhere (and briefly explain any exclusions if applicable).  • Sampling & response rate: Describe sampling approach (consecutive? convenience? systematic?) and report response rate (invited vs enrolled), per checklist.  • Measurement validity & reliability: Add validity/translation information for Rosenberg Self-Esteem Scale (RSES) in this population (e.g., cultural/linguistic adaptation, Cronbach’s alpha in your sample). This is currently missing and required by the checklist.  • Variable definitions: You defined low self-esteem as RSES < 15—state rationale with citation.  • Statistical analysis: You stated modified Poisson with robust SEs and p≤0.20 for entry—good. Add specifics: which covariates entered a priori vs. data-driven; handling of missing data; checks for multicollinearity; model fit diagnostics (if any).  • Qualitative methods: Nice detail (IDIs/KIIs, language, transcription, coding, saturation). Add reference or short description of the THRIVE framework and clarify why it guided analysis.  • Ethics/consent: Approval number is present; include approval date and specify written consent/assent clearly (parents/guardians vs 18–19y participants) inside Methods (not only in front matter), per checklist.  5) Results  • Participant characteristics:  o Wording check: “Most (58.5%) had no formal education.” For adolescents 10–19, this is unusual; likely means “no secondary education” or “not beyond primary.” Ensure accuracy of the label and interpretation. The table shows “None, Primary, Secondary, Tertiary”—verify data coding, then rewrite text to match actual schooling stages.  • Tables: numbering, captions, and footnotes:  o Tables jump (1, 3, 4, 7). Ensure a continuous sequence (Table 1, 2, 3, …).  o Title format per checklist: include participants, place, and time (e.g., “Baseline characteristics of adolescents with SCD attending MNRH SCC, Kampala, Uganda (Jan 8–Mar 26, 2024), N=356”). No full stop at the end of table titles.  o Standardize column headers to “No. (%)” and remove “%” from cell entries.  o Keep totals to exactly 100% (round carefully; add footnote if rounding causes ±0.1%).  o Use asterisks solely for significance; add footnotes to explain symbols.  o Per checklist, report p-values only (omit test statistics like χ² in tables).  • Bivariate text accuracy: The narrative says “and so were taking hydroxyurea” among factors associated with LSE, but the table shows PR = 0.92, p = 0.092 (not significant). Remove that claim or qualify it as non-significant.  • Multivariable model wording: Present contrasts clearly (what is reference category; write “Compared with those frequently absent from school, adolescents not frequently absent had lower prevalence of LSE (aPR…)).” Ensure the narrative matches the direction of the coefficients you present.  • Figure(s): If any figures exist in the source files, ensure legends are complete (participants, place, time). If none, consider a single adjusted PR forest plot for the three key variables to improve clarity (optional but helpful).  6) Qualitative results  • Framework & themes: Clear five-domain synthesis consistent with THRIVE; quotes are appropriate.  • Attribution: Keep anonymized age/sex/grade labels consistent; ensure all quotes are translated and indicated as such when not originally in English.  7) Discussion  • Start with key findings (no mini-introduction).  • Avoid repetition of numerical results. Interpret directions: social limitation ↑LSE prevalence; school presence and physical ability ↓LSE prevalence.  • Literature consistency:  o Fix author name inconsistencies used in-text: you cite “Forrestal” in prose but the reference lists “Forrester AB”—standardize.  o “Siegel/Seigel” spellings differ between text and references—standardize.  • Strengths & limitations: Move Limitations to its own subsection (see next section) and avoid exaggeration in Strengths.  8) Limitations (separate subsection “4.1 Limitations”)  Create a brief, explicit subsection that discusses:  • Cross-sectional design (no causal inference).  • Potential measurement bias (RSES cultural validity; social desirability).  • Possible misclassification of schooling level and activity limitations.  • Clinic-based sample (generalizability).  • Missing data handling (if any).  This is specifically required by the checklist.  9) Conclusion  • Keep it brief and answer the aim directly, without references. Add one-sentence practical implication (e.g., integrate psychosocial screening and activity-friendly guidance in clinic SOPs).  10) Ethics, data availability, funding, competing interests  • Ethics statement: Add the approval date and keep the approval code; ensure this exact text also appears within Methods.  • Data availability: Statement says “All relevant data are within the manuscript and its Supporting Information files.” Ensure the Supporting files link(s) are live at submission and mention file names/types in the statement.  • Funding: Provide funder details per PLOS format (initials, grant number, funder name, URL, and the no-role statement). Your draft text is close; just ensure initials are included for recipients and add the funder URL(s).  • Competing interests: “The authors have declared that no competing interests exist.” Fine.  11) Language and style  • Voice: Checklist prefers first person over heavy passive constructions (e.g., “We conducted… We used…”).  • Consistency: Use “adolescents with SCD” throughout; avoid mixing “children/teenagers” unless defined.  Spelling/typos: Fix “Thrive”→“THRIVE”; “college of health sciences”→“College of Health Sciences”; “Mulago national referral hospital”→“Mulago National Referral Hospital”; “Ndeezi/Ndezi” consistency.  12) References (Vancouver, with DOIs/URLs)  • Vancouver compliance: Re-format all references strictly to Vancouver style with abbreviated journal titles.  • Correct author names & titles: Align in-text citations with the reference list (Forrester vs Forrestal; Siegel vs Seigel; Pediatric Blood & Cancer capitalization).  • Add DOIs for journal articles wherever available; for websites include URL and access date.  • Count: ~40 is typical for an original article; your count is within range but finalize after deduplication and corrections. | Title revised as recommended and we appreciate the guidance offered on revisions of the title page. (Line 3)  Affiliations revised as recommended (line 7-8)  Correct name is Grace Ndeezi and this has been Revised-Line 5  All sections have been numbered  This has been rewritten as guided to provide direction as guided(abstract) and multivariate results section  Introduction has been revised as guided, the use of THRIVE has been capitalized throughout the text, the purpose of the study appears as the last sentence and duplicate statements have been removed. We are very grateful for highlighting the comments raised.  We appreciate the editor for pointing out the issues raised. The Materials and methods section has been revised as guided. Sample size is 356 consistently throughout the text,  Sampling has been revised to include approach. Line 103-107    Unfortunately, were not able to pretest the data collection data, however the tool has demonstrated robustness among adolescents in sub Saharan African with two referenced studies demonstration Cronbach alpha of 0.7-0.8. Line 134-137  Statistical analysis has been revised, details on collinearity, handling of missing data have been added. Line 149-157  A reference has been added and also a justification for using the thrive framework  Line 213-216  This has been addressed as guided  Thank you for this observation. In our dataset, the category “no formal education” was used to refer to adolescents who had **not completed primary education**. We agree that in the context of adolescents aged 10–19 years, this terminology may be misleading. To improve clarity, we have revised the wording in the manuscript to state **“had not completed primary education”** instead of “no formal education.” The interpretation in the results and discussion has been updated accordingly.  This was an oversight ad we thank the academic editor for pointing it out. The tables have been sequentially numbered and titles have been corrected  This has been revised as guided and all total add up to 100%  We thank the editor for pointing this out. Only P values and measure of association are reported in this study.  Yes, at bivariate significance is at 0.2 so taking hydroxyurea with p =0.092 was associated with LSE at bivariate  Multivariate has been revised as guided such that narrative matches direction of coefficients. Line 199-208  We appreciate the editors concerns. Our study did not originally include any figures; we believe the key findings are already clearly presented in the tables  Age/sex.grade labels are consistent throughout the text  The mini introduction has been removed and the discussion starts with the results.  This has been rewritten to provide direction as earlier guided.  The spellings have been corrected  The limitations have been explicitly stated with all the possible forms of bias in the study.  Line 307-317  The conclusion has been kept brief as guided.  This is revised as guided.  The funding statement has been revised as guided.  390-391  We appreciated the editors concerns. The text has been revised throughout the texts as guided. |
|  | Reviewer 1 | |
|  | The study addresses a timely and important gap: mental health, specifically self-esteem, among adolescents with sickle cell disease (SCD) in a low-resource setting. The mixed-methods design is appropriate, and the integration of qualitative insights (example, coping strategies like prayer, peer support, and medication adherence) enriches the quantitative findings.  Here are some major methodological concerns that undermine technical soundness:  • Unconventional RSES cutoff: The authors define low self-esteem as a Rosenberg Self-Esteem Scale (RSES) score <15 (lines 30–31, 102–103). This is atypical. The standard RSES ranges from 0–30, and most studies (including in African contexts) use ≤25 or ≤20 to indicate low self-esteem. A cutoff of <15 suggests only the most severely affected are classified as having low self-esteem, which likely underestimates prevalence and may explain why the reported prevalence (21.6%) is lower than in comparable studies (e.g., 75–76% in U.S. and Congo; lines 260–262). The authors do not justify this threshold, nor cite validation studies supporting its use in Ugandan adolescents. This is a critical flaw that affects all downstream analyses and conclusions.  • Key predictors like “limited physical activity” and “limited social engagement” (lines 170, Table 1) are not operationally defined. How were these assessed? Were standardized tools used, or was this based on a single-item self-report? Without clarity, reproducibility and validity are compromised.  In Table 4, the reference group for school absenteeism is “Yes” (frequently absent), and the adjusted PR for “No” is 0.94. While statistically correct, this framing is counterintuitive and risks misinterpretation. It would be clearer to code “No absenteeism” as the reference so that PR >1 reflects increased risk. As written, readers may mistakenly infer that absenteeism is protective.  • Age range concerns: Including 10-year-olds (line 94) in an “adolescent” study using the RSES, a scale validated primarily for ages 12+, raises developmental validity questions. Young children may interpret items differently, potentially biasing their scores.  The use of modified Poisson regression to estimate prevalence ratios is appropriate for common outcomes (low self-esteem prevalence >10%). However:  • The manuscript states that variables with p ≤ 0.2 in bivariate analysis were eligible for multivariable modeling (lines 132–133). Yet, several such variables (e.g., hydroxyurea use, p = 0.092; hospitalization, p = 0.211) were excluded from the final model without justification (Table 3 vs. Table 4). This selective inclusion weakens transparency.  No model diagnostics (e.g., goodness-of-fit, multicollinearity checks) are reported. Given the small number of covariates, this is a missed opportunity to strengthen rigor.  •  Strengths  • The qualitative component is rich and contextually grounded, offering actionable insights (e.g., the role of faith, peer awareness, caregiver support).  • The focus on modifiable factors (school attendance, physical activity, social engagement) provides a foundation for interventions  .  This is a valuable and socially relevant study that sheds light on an under-addressed aspect of SCD care in Uganda. With methodological clarifications, particularly regarding the RSES cutoff and variable definitions, the manuscript could make a strong contribution to the literature. I recommend a major revision before acceptance. | We appreciate the reviewer’s concern regarding the RSES cut-off. While the original Rosenberg scale does not prescribe a specific threshold, a number of published studies have used a score of ≤15 to classify low self-esteem; (we cite 2 such studies within the manuscript) therefore, our choice of this cut-off is not unique to our study. However, we acknowledge that this more conservative threshold may have contributed to an underestimation of the true prevalence of low self-esteem in our sample. In line with the reviewer’s recommendation, we acknowledge this limitation.  Line 112-114  Line 143  We thank the reviewer for this important observation. We agree that clearer operational definitions are necessary to ensure reproducibility. In the revised manuscript, we have now explicitly defined how “limited physical activity” and “limited social engagement” were assessed. These variables were measured using single-item self-report questions developed for this study based on routine clinical assessments conducted in the sickle cell clinic. No standardized instruments were used. We have clarified this in the Methods section and expanded the Limitations section to acknowledge the potential for misclassification and recall bias resulting from non-standardized, self-reported measures.  We thank you for this observation, the table of multivariate results has been revised as guided such that narrative matches direction of coefficients  We thank the reviewer for highlighting the developmental considerations regarding the use of the RSES among 10–11-year-olds. We acknowledge that the RSES has primarily been validated for adolescents aged 12 years and above, and younger participants may interpret some items differently. The inclusion of 10–11-year-olds was intentional because this age group constitutes a substantial proportion of children attending the sickle cell clinic and experiences similar psychosocial challenges. we have further strengthened the Limitations section to acknowledge the potential for developmental misinterpretation of RSES items in younger participants.  Line 362-265  We thank the reviewer for this important observation. In our analysis, all variables with p ≤ 0.20 at bivariate level were initially entered into the multivariable modified Poisson regression model as stated. We then applied a backward stepwise elimination approach, sequentially removing variables that did not contribute significantly to the model (p > 0.05) while assessing changes in model fit at each step. Variables such as hydroxyurea use and prior hospitalization did not retain statistical significance in the presence of other covariates and did not improve overall model performance; therefore, they were omitted from the final model. This has been explicitly stated in the revised manuscript.  Line 149-157  Thank you for this important observation. We agree that reporting model diagnostics is essential for demonstrating the robustness of the multivariable model. In the revised manuscript, we have now added details on the diagnostic checks performed. Specifically, we assessed **multicollinearity** using the Variance Inflation Factor (VIF), with all covariates showing VIF values <2, indicating no concerning collinearity  Line 155-157 |
|  | Reviewer 2 | |
|  | Overall, this is a well-written paper that handles an important discussion surrounding self-esteem in adolescents with sickle cell disease thoughtfully and skillfully. I have a few suggestions to improve the paper, as listed below:  - I would recommend re-reviewing the paper to ensure that all sentences are complete (e.g. line 70, lines 94-95).  - Table 1 has a category for Limited Social Engagement (91.6% reported "Yes"), while also separately reporting "Have family and peer support" (95.8% reported "Yes"). Would you please expand upon the differences between these two questions? This will also need explained further in the paper when the table is discussed.  - Line 186 mentions the THRIVE framework, but I did not see this explained elsewhere in the paper. Could you please define this/provide a reference for this, as it is not common knowledge?  - I would suggest adding a different heading prior to your section listing direct quotes from study participants to better frame that section of the paper. | Family support has since been deleted as variable due to collinearity.  A clear justification for using the thrive framework has been included. |
